# Gastric and colonic metastasis from NSCLC: A very unusual case report

**DOI:** 10.1097/MD.0000000000028249

**Published:** 2022-01-14

**Authors:** Martina Catalano, Andrea Marini, Katia Ferrari, Luca Voltolini, Fabio Cianchi, Camilla Eva Comin, Francesca Castiglione, Giandomenico Roviello, Enrico Mini

**Affiliations:** a School of Human Health Sciences, University of Florence, Largo Brambilla 3, Florence, Italy; b Respiratory Medicine, Careggi University Hospital, Florence, Italy; c Thoracic Surgery Unit, Careggi University Hospital, Largo Brambilla, 1, Florence, Italy; d Department of Experimental and Clinical Medicine, University of Florence, Largo Brambilla 3, Florence, Italy; e Department of Experimental and Clinical Medicine, University of Florence, Italy; f Histopathology and Molecular Diagnostics Unit, Careggi University Hospital, Florence, Italy; g Department of Health Sciences, Section of Clinical Pharmacology and Oncology, University of Florence, VialePieraccini, 6, Florence, Italy.

**Keywords:** gastrointestinal metastases, non-small cell lung cancer, primary lung cancer

## Abstract

**Rationale:**

Lung cancer is the most common cause of cancer-related deaths worldwide. Approximately 50% of patients is metastatic at diagnosis and the most common metastatic sites are bone, lungs, brain, adrenal glands, liver, and extra thoracic lymph nodes. The occurrence of gastrointestinal metastasis from lung carcinoma is rare and seems more commonly related to small cell lung cancer compared to non-small cell lung cancer (NSCLC).

**Patient information and diagnosis:**

A 78-year-old man with completely surgically resected NSCLC and no initial evidence of distant metastases developed colon and gastric metastases 7 months after diagnosis, confirmed by serial radiological examinations and endoscopic biopsies.

**Interventions:**

The patient was subjected to total gastrectomy with D2 lymph node dissection plus partial colectomy for intraoperative detection of a transverse colon neoformation. Subsequent instrumental imaging showed bilateral lung tumor recurrence, treated with gemcitabine monotherapy for 8 months as first line chemotherapy for lung adenocarcinoma.

**Results:**

The patient presented complete response to therapy and was disease-free for 4 years.

**Lessons:**

Colonic and gastric metastasis are very infrequent in NSCLC. The resection of gastrointestinal metastasis may provide benefits in terms of both symptom control and survival in patients properly selected.

## 1 Introduction

Lung cancer is one of the common malignant tumors with higher incidence and mortality, mainly divided in non-small cell lung cancer (NSCLC) and small cell lung cancer (SCLC), according to histopathological features. NSCLC accounts for about ∼85% of lung tumors and commonly presents metastasis at the diagnosis.^[1]^ The occurrence of gastrointestinal (GI) metastasis from lung carcinoma is rare (0.5%-10%).^[2]^ It is more commonly related to SCLC compared with NSCLC and frequently occurs in the small bowel.^[3]^ Gastrointestinal metastases may result in bleeding, obstruction, and/or perforation.^[4]^ Here, we report a rare case of a patient with NSCLC without evidence of distant metastasis and complete response to surgery, who developed gastric and colonic metastasis 7 months after the diagnosis.

## 2 Case report

**Figure 1:**
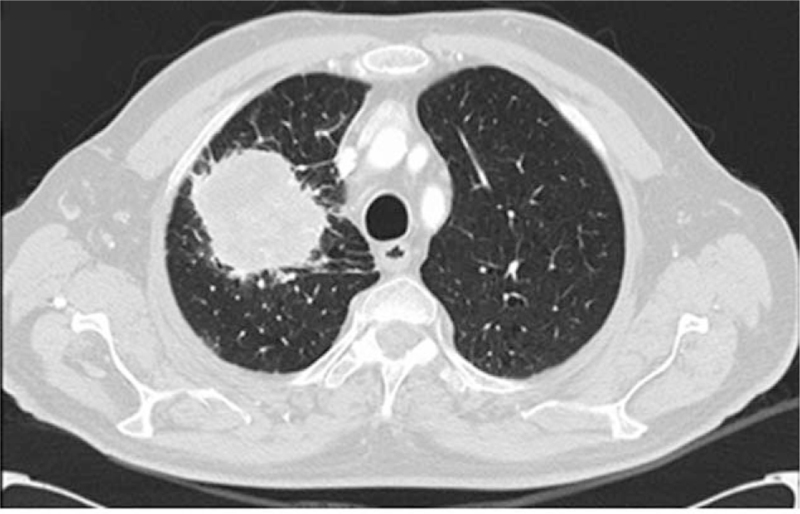
Computed tomography scan at diagnosis: right upper lung non-small cell lung carcinoma (NSCLC) (pT2bN0M0) of 6×6.5×4 cm.

**Figure 2:**
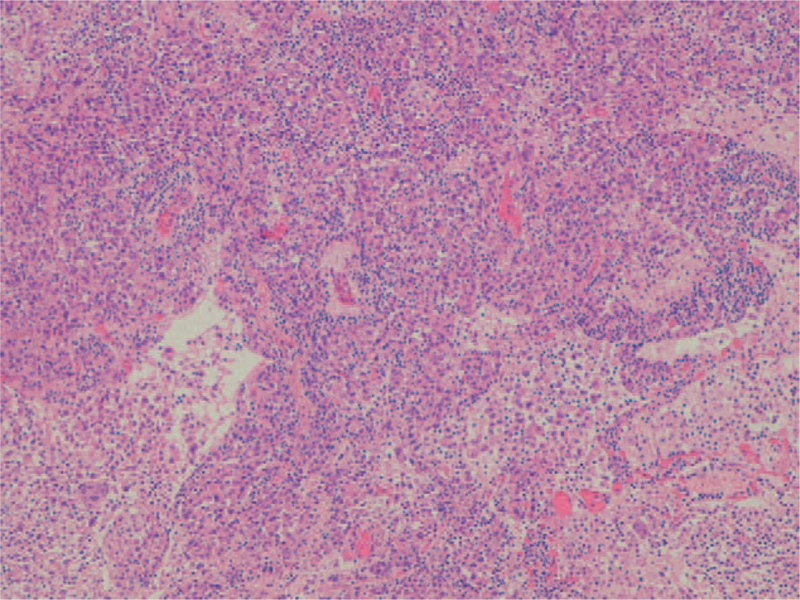
Primary lung adenocarcinoma (H&E ×10).

**Figure 3:**
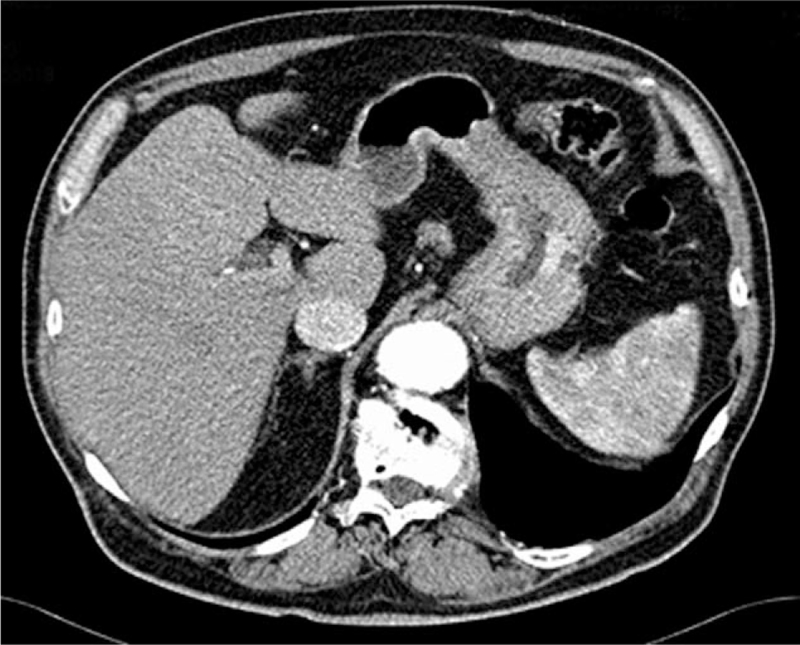
Computed tomography scan 5 months after surgery: gastric thickening at the greater curvature side of 1.5 cm in size.

**Figure 4:**
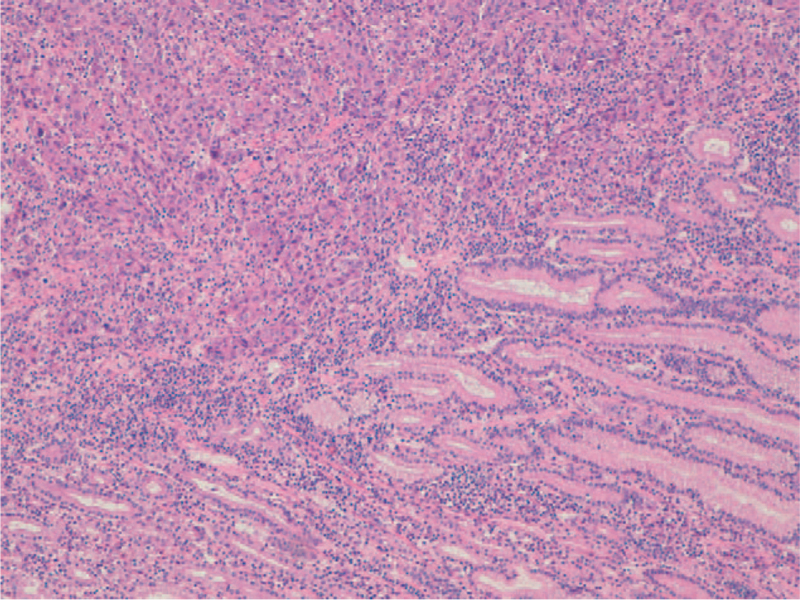
Lung adenocarcinoma infiltrating the gastric wall (H&E ×10).

**Figure 5:**
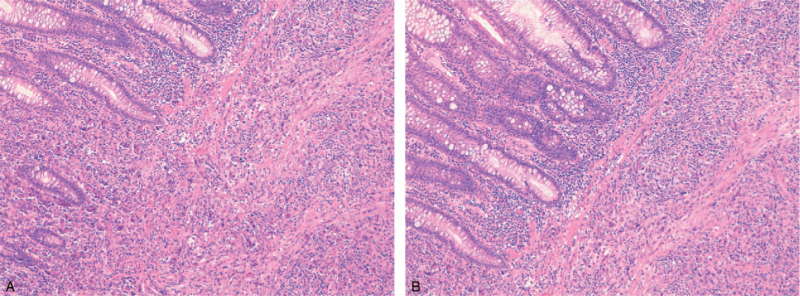
(A and B) Lung adenocarcinoma infiltrating the transverse colon (H&E ×10).

A 78-year-old man with a history of smoking (20 cigarettes *per* day for 40 years), was diagnosed with right upper lobe NSCLC (pT2bN0M0). The opening presentation included only cough and mild dyspnea for 4 weeks. The initial tumor size was 6×6.5×4 cm on computed tomography (CT) examination (Fig. 1). Fibro bronchoscopy was performed, and pathological examination revealed poorly differentiated NSCLC, thyroid transcription factor-1+, p63–. By means of whole-body CT examination, no evidence of distant metastasis in any sites, including the brain, had been evidenced. The patient underwent surgical treatment with upper right lobectomy and lymphadenectomy without any subsequent treatment. No documentable neoplastic proliferation in the resection margins and in the examined lymph nodes was found. Immunohistochemical staining confirmed the diagnosis of adenocarcinoma positive for TTF-1 and cytokeratin (CK) 7 and negative for p63 and CK5/6 (Fig. 2). The genetic profiling and the PD-L1 expression are reported in Table 1. A subsequent CT scan, 5 months after surgery, demonstrated 1.5 cm gastric irregularity in the stomach great curvature (Fig. 3), clinically asymptomatic. Upper gastrointestinal endoscopy showed a new formation projecting in the lumen and centrally eroded. The biopsy revealed adenocarcinoma CAM 5.2+; CK7+; periodic acid Schiff-diastase+. The patient was subjected to total gastrectomy with D2 lymph node dissection, cholecystectomy, and partial colectomy for occasional finding of a colon neoformation. No documentable neoplastic proliferation in the resection margins, in the 15-peri gastric lymph nodes and in the other examined tissues was found. Histologic examination showed a poorly differentiated adenocarcinoma engaging gastric wall and colon transverse. Immunohistochemical analysis revealed positivity for thyroid transcription factor 1, Napsin-A, CK7 and CAM5.2 and negative staining for CK20 and CDX-2. Peri-gastric lymph nodes were negative for metastasis (Figs. 4 and 5A, B). The molecular profiling is reported in Table 1. Surgery was complicated with infective pneumoniae requiring hospitalization and resolved with antibiotic therapy. Two months after metastasis surgery, computerized tomography imaging showed an excavated lesion in the right lower lobe and a nodular lesion of 4 mm in the left upper lobe. The patient received a total of 11 cycles of gemcitabine 1000 mg/m^2^ administered intravenously on days 1, 8 every 3 weeks, 2 weeks after metastasis resection. Subsequent constant controls did not show disease relapse and the patient was disease-free for 4 years. In February 2021 during follow-up checks with total body CT, the appearance of a swelling of the left adrenal gland with some pseudo nodular inhomogeneities the greater of 2.2 cm in the same context and a 1.8 cm lymph adenomegaly in the left antero-lateral lumbo-aortic area was highlighted. Biopsy performed on the adrenal gland nodule confirmed the pulmonary origin of the secondary lesion. According to the metastatic lung cancer guidelines, a second-line therapy with pembrolizumab (humanized antibody directed against programmed death-1 receptor) has been initiated and is still ongoing.

**Table 1:** Genetic profile and PD-L1 expression of primary lung cancer and gastrointestinal metastases.

Primary lung cancer		Gastrointestinal metastases
70%	PD-L1 expression	90%
Negative	EGFR mutations	Negative
Negative	ALK rearrangements	Negative
Negative	ROS 1 rearrangements	Negative
Negative	Molecular profiling ALK, BRAF, EGFR, ERBB2, FGFR3, HRAS, IDH1, IDH2, KIT, KRAS, MET, NRAS, PDGFRA, PIK3CA, RET, ROS1	Negative
N/A	NTRK fusion	Negative

ALK = anaplastic lymphoma receptor tyrosine kinase, EGFR = epidermal growth factor receptor, ERBB2 = human epidermal growth factor receptor 2, FGFR3 = fibroblast growth factor receptor 3, IDH = isocitrate dehydrogenase genes, PDGFRA = platelet derived growth factor receptor alpha, PD-L1 = Programmed death cell ligand 1, PIK3CA = phosphatidylinositol-4,5-bisphosphate 3-kinase catalytic subunit alpha, ROS-1 = ROS proto-oncogene 1.

## 3 Discussion

The most common NSCLC metastatic site is bone (34%), followed by lungs (32%), brain (28%), adrenal glands (17%), liver (13%), and extra thoracic lymph nodes (9%). Lung cancer metastasis to the gastrointestinal tract are rare (0.5%-10%),^[2]^ and most commonly occur in the small bowel.^[3]^ Colonic metastases is uncommon with an incidence of 0.1% as reported.^[5]^ Likewise, gastric metastasis is rare with an incidence to range between 0.2 and 1.7% as reported by autopsy data.^[6]^ Overall, only 44 cases of lung cancer metastasized to the colon have been published so far.^[5]^ Lin et al^[7]^ reported 4 gastric and 2 colon metastasis among 18 patients with lung cancer and GI metastasis. In Fujiwara et al^[8]^ study on 1552 patients with NSCLC who received surgical treatment, only 9 (0.58%) patients showed GI metastasis. Among them, 4 patients had colonic metastasis and only 1 patient had gastric metastasis. Huang et al^[9]^ reported the first case of primary lung adenocarcinoma with both gastric and colonic metastasis at the diagnosis, accompanied by peritoneal carcinomatosis and multiple active bone lesions.

As reported in several studies, squamous cell carcinoma is the first histology related to colon metastasis,^[6]^ followed by adenocarcinoma and SCLC.^[6]^ It is still controversial if gastric metastases, are most correlated with SCLC subtype.^[10,11]^ or lung adenocarcinoma.^[6]^

Lung cancer with gastric metastasis seems to occur mainly in male smokers aged between 45 to 90 years.^[12–14]^ It has been hypothesized that the gastrointestinal metastases are due to sputum swallowing rich in cancer cells that reaches the digestive tract; this mechanism is especially relevant in smokers who are more susceptible to gastric mucosal damage than nonsmokers.^[15]^

An analysis on the gastric metastasis from solid malignant tumors has revealed that they mimic submucosal tumors in the 52% patients and primary gastric cancers in the 39% of patients^[16]^; in the second case occur as bull's eye signs, volcano-like ulcers, or surface umbilication.^[12,17–19]^ An infiltrating “linitisplastica” pattern has been seen in only 2% of cases in lung cancer.^[20]^

The most frequent initial clinical symptom of metastatic colon cancer was abdominal pain due to intestinal tract obstruction, following by bloody stool due to either melena or hematochezia.^[5]^ Metastatic colonic neoplasms from lung cancer can also present initially with non-bloody diarrhea, encopresis, and hyponatremia.^[21,22]^ Most gastric metastasis from primary lung cancer are asymptomatic and often discovered during autopsy^[18]^ due to the usually implant in the gastric submucosa. Nonspecific epigastric pain and chronic bleeding resulting in melena and anemia are common clinical manifestations in symptomatic patients.^[12]^ Perforation and acute bleeding are rare but related with high mortality.^[3,13]^ Due to the low incidence and limited information regarding typical symptomatology, the initial diagnosis of colonic and gastric metastasis from lung carcinoma is challenging.

Histological examination, in correlation with clinical findings, remains the gold standard for diagnosis; immunochemistry assists in identifying the origin of the primary tumor. Immunochemistry stains such as TTF-1, CDX2, CK7, CK20, CK-14, and CK-18 can help distinguish metastatic lung carcinoma from primary gastrointestinal cancer.^[12,23,24]^ Positive staining for CK-7is consistent with either gastrointestinal or pulmonary origin, whereas CDX2 expression suggest gastrointestinal origin. Positive staining for CK-14 and CK-18 can be related to squamous cell carcinoma and adenocarcinoma, respectively. TTF-1 regulates gene expression in the thyroid, lung, and diencephalon during embryogenesis.^[25]^ TTF-1 appears to be helpful in distinguishing tissues of pulmonary origin,^[26,27]^ although some data indicate that 13% to 45% of metastatic adenocarcinomas of pulmonary origin are TTF-1 negative.

Napsin-A, a functional aspartic proteinase expressed in the cytoplasm of healthy lung parenchyma, is more sensitive than TTF-1 in distinguishing primary lung carcinoma from other adenocarcinomas.^[25–28]^ Indeed, it is a useful additional immunohistochemical staining to TTF-1 for determining the origin of metastatic adenocarcinomas. Furthermore, unlike other conventional instrumental examinations, positron emission tomography (PET) -TC could favor the differentiation between the primary and secondary origin of the tumor without being able to establish the histopathological type of the tumor cells.^[27,29,30]^

Optimal management of gastrointestinal metastases from primary lung cancer is controversial. Chemotherapy is the main option in the recurrent or metastatic NSCLC treatment. However, chemotherapy induced necrosis could increase the risk of gastric bleeding or perforation as has been previously reported.^[31]^ Lee et al^[32]^ described longer survival in patients with gastric and/or duodenal metastases managed with supportive treatment without surgery. Conversely, aggressive surgical treatment for NSCLC with gastric metastasis, including lobectomy for primary pulmonary tumors plus lymphadenectomy and gastrectomy for gastric metastasis have been described.^[11,15]^

In Fujiwara et al,^[8]^ 5 patients underwent gastrointestinal surgery, and 3 patients have experienced long survivals after resection without recurrence; in the other 2 patients gastrointestinal symptoms were well controlled after surgery. In contrast, 4 patients who did not undergo surgery had a shorter survival after the diagnosis of gastrointestinal metastasis. Radical resection of isolated GI metastasis seems to be advantageous although a small number of cases have been reported but the clinical impact on long-term outcome of surgical resection of a GI metastasis remains to be clarified. However, surgery is still necessary to prevent life-threatening complications such as massive hemorrhage, obstruction and perforation thus providing effective palliation.^[32,33]^ In the present case, surgical intervention was performed with curative intent as the patient had no GI symptoms.

Average overall survival of patients with primary lung carcinoma, from the diagnosis of gastrointestinal metastasis to death, is quite variable. For colonic metastasis has been reported to be approximately 2 months.^[2,30]^ Kim et al^[29]^ reported that the median survival time in pulmonary carcinoma with gastrointestinal metastasis was 94.5 days, ranging from 12 to 1907 days. Outcomes are based on main events at the time of initial presentation and subsequent surgical intervention. Perforation, obstruction, or hemorrhage have been associated with less favorable outcomes.^[5]^ In reverse, early detection and surgical intervention as well as palliative surgical resection of the metastatic site have been assumed to improve survival.^[2,29,34]^

The unusually good clinical outcome in our patient, although the presence of a double metachronous metastases, could be explained by the early diagnosis of the metastasis, the absence of GI signs or symptoms and good performance status of the patient. Therefore, resection of gastrointestinal metastasis may provide benefits in terms of both symptom control and survival in patients properly selected.

## 4 Conclusion

Gastrointestinal metastases from lung cancer are rare and less commonly reported in NSCLC than SCLC. The contemporary presence of both gastric and colonic metastasis from lung cancer is even more unusual. Radiological and endoscopic examinations are clinically useful for establishing a diagnosis, but pathological diagnosis is mandatory. Immunohistochemical staining with Napsin-A and TTF-1 may help differentiating gastric metastases from primary gastric cancer. More data on GI metastasis from lung carcinoma are required to clarify clinical features and outcomes. Nevertheless, surgical resection should be considered in patients with intractable bleeding, obstruction, or perforation but also be considered in those with resectable primary lung cancer and isolated gastrointestinal metastasis as it seems to improve survival outcome.

## Author contributions

**Conceptualization:** Francesca Castiglione.

**Funding acquisition:** Martina Catalano.

**Investigation:** Martina Catalano, Katia Ferrari.

**Methodology:** Martina Catalano, Francesca Castiglione.

**Project administration:** Giandomenico Roviello.

**Resources:** Giandomenico Roviello.

**Supervision:** Camilla Eva Comin, Giandomenico Roviello, Enrico Mini.

**Validation:** Fabio Cianchi, Camilla Eva Comin, Giandomenico Roviello.

**Visualization:** Luca Voltolini.

**Writing – original draft:** Martina Catalano, Andrea Marini.

**Writing – review & editing:** Martina Catalano.
